# Supplementary material for: The biased hand. Mouse-tracking metrics to examine the conflict processing in a race-implicit association test
Source: PLoS One. 2022 Jul 27;17(7):e0271748. doi: 10.1371/journal.pone.0271748 (PMC9328548; doi:10.1371/journal.pone.0271748)
Supplement: S1 File — (DOCX) [file pone.0271748.s001.docx]

**Supporting Information**

**The biased hand. Mouse-Tracking metrics to examine the conflict processing in a race-implicit association test**

Michael Di Palma^1*^, Desiré Carioti^2^, Elisa Arcangeli^2^, Cristina Rosazza^2^, Patrizia Ambrogini^3^, Riccardo Cuppini^3^, Andrea Minelli^3^, Manuela Berlingeri^2,4,5*^

^1^ Department of Clinical and Experimental Medicine, Università Politecnica delle Marche, Ancona, Italy

^2^ Department of Humanities, University of Urbino Carlo Bo, Urbino, Italy

^3^ Department of Biomolecular Sciences, University of Urbino Carlo Bo, Urbino, Italy

^4^ NeuroMi, Milan Centre for Neuroscience, Milan, Italy

^5^ Center of Developmental Neuropsychology, Area Vasta 1, ASUR Marche, Pesaro, Italy

***Corresponding author**

E-mail: m.dipalma@staff.univpm.it (MDP)

E-mail: manuela.berlingeri@uniurb.it (MB)

**Supporting Table 1.** Participant characteristics. EHI = Edinburgh Handedness Inventory.

|  | **Male participants** | | | **Female participants** | | | **Wilcoxon rank-sum test** | | |
| --- | --- | --- | --- | --- | --- | --- | --- | --- | --- |
|  | **Median** | **Min** | **Max** | **Median** | **Min** | **Max** | ***W*** | ***r*** | ***p*** |
| **Age** | 23 | 19 | 32 | 22 | 18 | 35 | 1401.5 | -0.129 | 0.164 |
| **Handedness Index** | | | | | | | | | |
| EHI | 85 | 35 | 100 | 86.67 | 35 | 100 | 1683.5 | -0.017 | 0.853 |


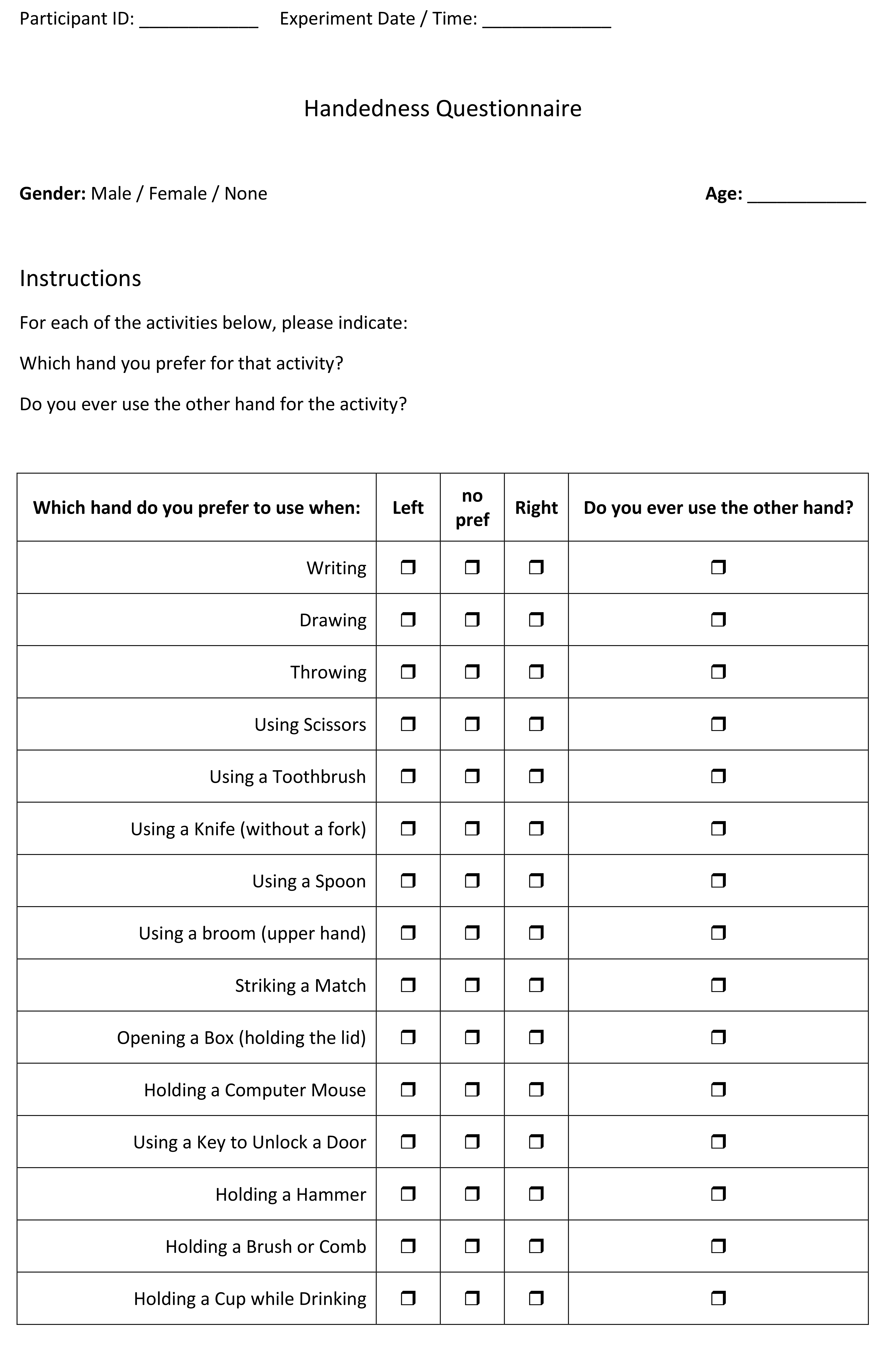


**Supporting Figure 1.** The ad-hoc created questionnaire administered to all participants at the beginning of the experimental session to assess participant’s handedness [1, 2].

**Supporting Table 2.** RT-race-IAT D Score.

| **RT-race-IAT D Score** | **Mean** | | **SEM** | | | **Min** | | | **Max** | | **One sample Student’s t-test** | | | |
| --- | --- | --- | --- | --- | --- | --- | --- | --- | --- | --- | --- | --- | --- | --- |
|  |  |  |  |  |  |  |  |  |  |  | ***H0:μ*** | ***t, df*** | | ***p*** |
| **Participants** | 1.08 | | 0.04 | | | 0.13 | | | 2.44 | | 0 | 26.43, 115 | | 0.0001** |
| **Male vs Female** | **Male participants** | | | | | **Female participants** | | | | | **Unpaired Student’s t-test** | | | |
|  | **Mean** | **SEM** | | **Min** | **Max** | **Mean** | **SEM** | **Min** | | **Max** | ***t, df*** | | ***p*** | |
|  | 1.13 | 0.06 | | 0.13 | 2.44 | 1.05 | 0.05 | 0.28 | | 1.95 | 1.05, 114 | | 0.293 | |
|  |  |  |  |  |  |  |  |  |  |  |  |  |  |  |

**The model syntax used for the MT-race-IAT congruency effect and conflict processing phases analyses.** A series of step-wise general linear effect models were adopted to assess the race-IAT Congruency Effect using the most representative observed variables of geometrical and temporal dimensions [i.e., Maximum Deviation (MD) as the observed variable for the geometrical factor and the Time when the Maximum Acceleration occurred (Acc-Max-Time) for the temporal factor]. The variables progressively involved in the model as fixed predictors were Type of attribute (Negative vs. Positive), and Race (Blacks vs. Whites), while the Subject was considered as clustering factor to model random intercept.

Accordingly, the basic structure of the estimated models to assess the MT-race-IAT Congruency Effect was built using the following syntax:

*M0 = lmer (Target observed variable ~ (1|Subject), data = mydata)*

*M1 = lmer (Target observed variable ~ Type of attribute + (*1*|Subject), data = mydata)*

*M2 = lmer (Target observed variable ~ Type of attribute + Race + (*1*|Subject), data = mydata)*

*M3 = lmer (Target observed variable ~ Type of attribute * Race + (*1*|Subject), data = mydata)*

Once computed the three phases model to temporally dissect the MT-race-IAT Congruency Effect, the between-phase differences in the amount of conflict generated in the stereotype-incongruent vs. stereotype-congruent trials of the MT-race-IAT task were explored using a general linear model with the Type of Phase (Early, Mid, Late) as fixed predictor and the Subject as random intercept. The basic structure of the estimated models was built using the following syntax:

*M0 = lmer (Congruency Effect ~ (1|Subject), data = mydata)*

*M1 = lmer (Congruency Effect ~ Type of Phase + (*1*|Subject), data = mydata)*

**Supporting Table 3.** Interclass Correlation Coefficient for Subjects. These indexes were adopted to identify the clustering factor that has to be considered in modelling the random intercepts. ICC = the Intraclass Correlation Coefficient, LowerCI = the Lower Confidence Interval limit, UpperCI = the Upper Confidence Interval limit, N = the total Number of individuals used in the analysis, K = the number of measurements per individual [3].

|  | **ICC** | **LowerCI** | **UpperCI** | **N** | **k** |
| --- | --- | --- | --- | --- | --- |
| **Intraclass Correlation Coefficient** | | | | |  |
| MD | 0.4166004 | 0.3209677 | 0.5159044 | 116 | 4 |
| Acc-Max-Time | 0.2878203 | 0.1938713 | 0.3911714 | 116 | 4 |
| Congruency Effect | 0.1778614 | 0.0104958 | 0.0276004 | 116 | 3 |

**The model syntax used for simple regressions.** The Δ-MD and Δ-Acc-Max-Time values were included in a General Linear Model (GLM) comprising the RT-race-IAT D score as a continuous independent variable using the following syntax:

*M0 = glm (Δ-MD ~ RT-race-IAT D score, data = mydata)*

*M1 = glm (Δ-Acc-Max-Time ~ RT-race-IAT D score, data = mydata)*

Here it is worth noting that before the GLM model the Cook’s distance was computed to identify outliers (see the following figure).


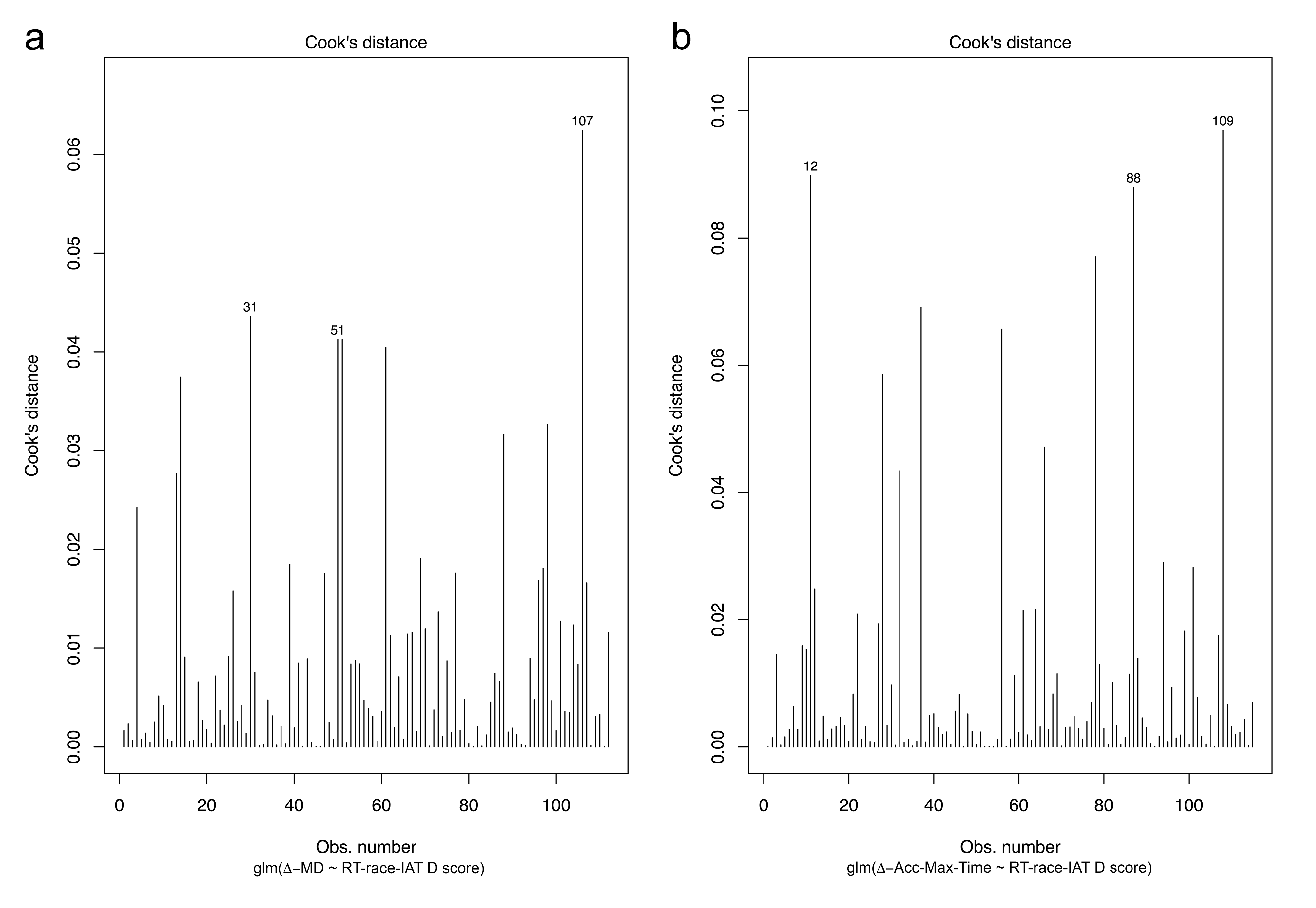


**Supporting Figure 2.** Cook’s distance Δ-MD (a) and Δ-Acc-Max-Time (b).

**Supporting Table 4.** Δ-MD and Δ-Acc-Max-Time regressions report.

| **Model** | ***Equation*** | ***b*** | ***SE b*** | **95% CI** | | ***R^2^*** | ***t*** | ***p*** |
| --- | --- | --- | --- | --- | --- | --- | --- | --- |
|  |  |  |  | ***LL*** | ***UL*** |  |  |  |
| Δ-MD ~ RT-race-IAT D score | Y = 35.73*X + 25.59 | 35.73 | 10.43 | 15.06 | 56.40 | 0.1 | 3.42 | < .001 |
| Δ-Acc-Max-Time ~ RT-race-IAT D score | Y = 44.97*X + 84.13 | 44.97 | 14.61 | 16.01 | 73.91 | 0.09 | 3.08 | .002 |
| SE = Standard Error; CI = confidence interval; LL = lower limit; UL = upper limit. | | | | | | | | |

The Early, Mid and Late values were included in a GLM with the RT-Race-IAT D score as a continuous independent variable using the following syntax:

*M0 = glm (Early Phase ~ RT-race-IAT D Score, data = mydata)*

*M1 = glm (Mid Phase ~ RT-race-IAT D Score, data = mydata)*

*M2 = glm (Late Phase ~ RT-race-IAT D Score, data = mydata)*

Here it is worth noting that before the GLM model the Cook’s distance was computed to identify outliers (see the following figure).


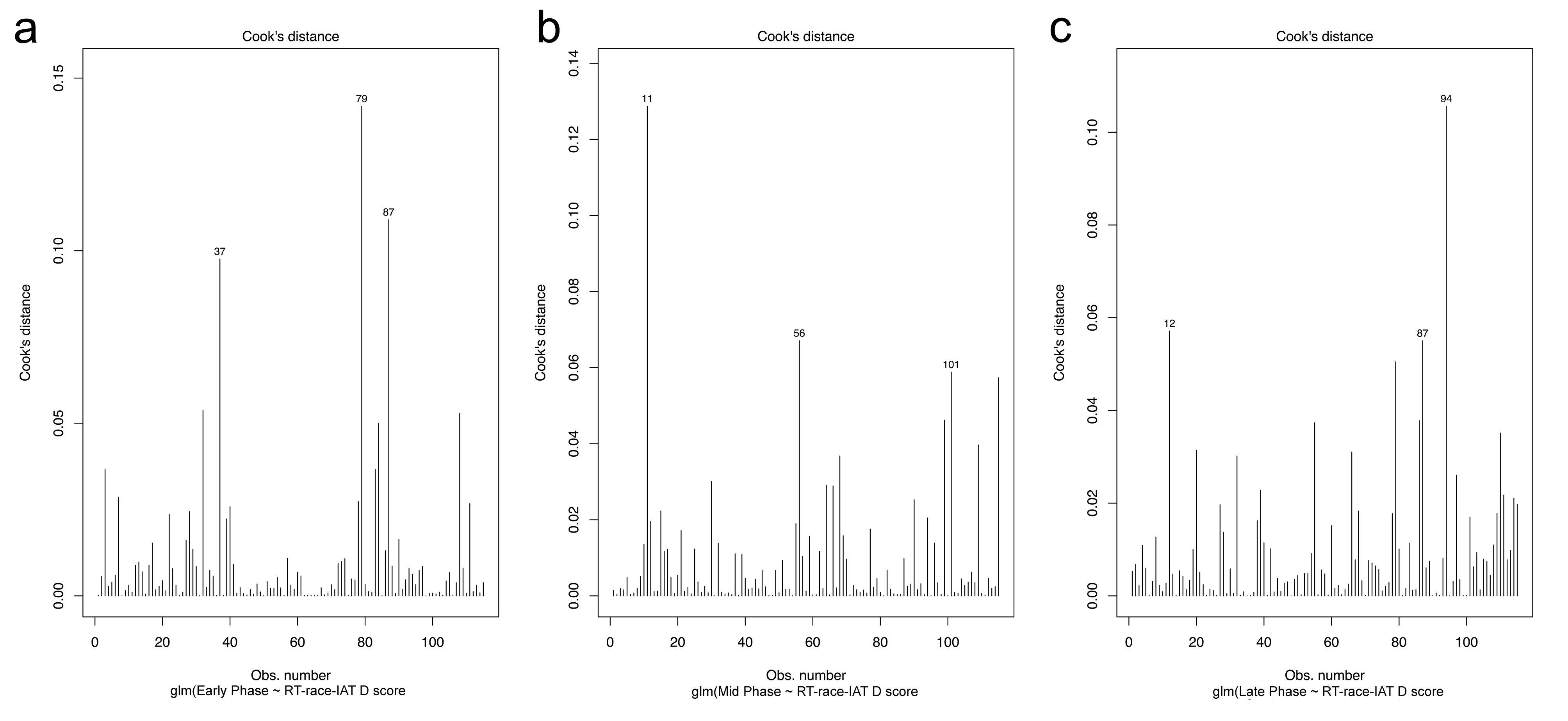


**Supporting Figure 3.** Cook’s distance Early (a), Mid (b) and Late (c) phase.

**Supporting Table 5.** Early, Mid and Late regressions report.

| **Model** | ***Equation*** | ***b*** | ***SE b*** | **95% CI** | | ***R^2^*** | ***t*** | ***P*** |
| --- | --- | --- | --- | --- | --- | --- | --- | --- |
|  |  |  |  | ***LL*** | ***UL*** |  |  |  |
| Early Phase ~ RT-race-IAT D score | Y = 30.29*X − 1.01 | 30.29 | 10.93 | 8.63 | 51.95 | 0.07 | 2.77 | .006 |
| Mid Phase ~ RT-race-IAT D score | Y = 27.83*X + 59.89 | 27.83 | 12.07 | 3.91 | 51.75 | 0.05 | 2.30 | .023 |
| Late Phase ~ RT-race-IAT D score | Y = 10.36*X + 28.40 | 10.36 | 10.35 | -9.92 | 30.64 | 0.008 | 1.00 | .318 |
| SE = Standard Error; CI = confidence interval; LL = lower limit; UL = upper limit. | | | | | | | | |

**Supporting References**

1. Oldfield RC. The assessment and analysis of handedness: The Edinburgh inventory. Neuropsychologia. 1971;9(1):97-113. doi: <https://doi.org/10.1016/0028-3932(71)90067-4>.

2. Cohen MS. Handedness questionnaire. Retrieved from <http://wwwbrainmappingorg/shared/Edinburghphp>. 2008.

3. Lessells CM, Boag PT. Unrepeatable Repeatabilities: A Common Mistake. The Auk: Ornithological Advances. 1987;104(1):116-21. doi: 10.2307/4087240 %J The Auk: Ornithological Advances.
