# Supplementary material for: The biased hand. Mouse-tracking metrics to examine the conflict processing in a race-implicit association test
Source: PLoS One. 2022 Jul 27;17(7):e0271748. doi: 10.1371/journal.pone.0271748 (PMC9328548; doi:10.1371/journal.pone.0271748)
Supplement: Attachment — Submitted filename: Di Palma et al - Response to Reviewers.docx [file pone.0271748.s002.docx]

First of all, we would like to thank the Reviewers for appreciating the methodology used in our work, and for all the efforts they made in trying to improve it. In what follows we address every single Reviewers’ issue and comment and we report the changes made into the revised version of the manuscript.

**Reviewer #1**

1. This study presents results of original research investigating conflict processing in a race IAT (Caucasian/African; positive/negative) among Caucasian native Italian speakers. Participants completed both a classical, reaction-time based race IAT and a mouse-tracking (MT) version. The manuscript is overall well-written, although I would like the authors to perform additional proofreading as several typos and minor errors are left in the manuscript.

*As suggested, we performed additional proofreading, and all the typos and minor language errors have been corrected. Accordingly, all the revisions had been highlighted in yellow to be easily detected.*

2. 120 participants took part in the study (a few excluded) and the authors seem to suggest that sample size was determined based on previous research using a MT version of the IAT. Although this rule of thumb may be sometimes warranted, I would have expected a more stringent a priori power analysis.

*We thank the Reviewer for the comment about the a-priori power analysis. In the original manuscript, we did not include the details about power-calculation, notwithstanding the Ethics Board of the University of Urbino always requires it. As the Reviewer can see from the details reported in what follows, we computed the sample size using the G*Power3 Software (http://www.gpower.hhu.de/) and taking the details from two studies incorporating MT-measures in an IAT structure (Yu et al., 2012; Smeding et al., 2016).*

***Figure 1.*** *Parameters employed for the sample size computation. Notably, the IAT-effect eta-squared (η^2^) reported on the work by Yu and coworkers (2012) was adopted for the Effect Size estimation. The Effect Size was estimated based on Cohen’s Method (Cohen, 1988) as implemented on G*Power software.*

***Figure 2.*** *Graphical representation of the non-centrality distribution for the critical F identification obtained using the power analysis parameters reported above.*

***Figure 3.*** *Sample size variation based on the desired statistic power. The data are plotted concerning the power analysis parameters reported above.*

*As shown in figure 3, the recruitment of 24 subjects would have allowed us to obtain a robust statistic power with a relatively conservative confidence level (1-α = 0.99). However, the sample size was increased to perform a Principal Component Analysis (PCA) on each condition of the MT-race-IAT (White-Positive, White-Negative, Black-Positive, Black-Negative), including all metrics collected. According to established guidelines for factor analysis (Gorsuch, 1983, p.332; Osborne and Costello, 2004), the subjects/variables ratio should be at least equal to 5:1 for attenuating potential errors and optimising generalizability of the PCA findings. This is the reason why we choose to test 120 participants.*

*We do apologise for omitting the power analysis details in the methods section. In the revised version of the manuscript, we have rephrased the inaccurate sentences on page 7 (rows 171-4) as follows:*

*‘Based on recent studies incorporating MT-measures in an IAT structure [31, 32], an a priori analysis with G*Power3 [43] was performed to estimate the sample size needed to explore with large effects size the race Congruency Effect. Accordingly, the recruitment of 24 subjects would have allowed obtaining a robust statistic power with a relatively conservative confidence level (f = 0.45, 1-β = 0.80, α = 0.001). However, the sample size was increased to perform a Principal Component Analysis (PCA) on each condition of the MT-race-IAT (White-Positive, White-Negative, Black-Positive, Black-Negative), including all metrics collected. Indeed, according to established guidelines for factor analysis [44, 45], the subjects/variables ratio has to be at least equal to 5:1 for attenuating potential errors and optimising the generalizability of the obtained findings. Therefore, the sample size was incremented from 24 to 120 (57.5% female)’.*

3. Given the theoretical background mobilized by the authors, I was wondering whether it is appropriate to state that "motor corrections [are] needed to connect cognition to action" -> this may (artificially) distinguish action from cognition, while we may consider the two are so intrinsically connected that this distinction is difficult to defend. What do the authors think?

*We see the Reviewer’s point of view, but we would like to better explain our statement. Indeed, the Reviewer may want to note that the whole reasoning, and not just an extracted part, containing the reported words in the manuscript at page 5 (rows 113-6) states:*

*‘Several neuropsychological works support using of hand movement as a proxy to detect and quantify the intertwined bottom-up and top-down strategic motor corrections needed to connect cognition to action during the decision process [17-20].’*

*Thus, in our opinion, the above adjective ‘intertwined’ stresses the intrinsic connection between cognition and action and that considering the whole reasoning, no artificial distinction between these functions is made.*

*However, taking into account the Reviewer criticism regarding the potentially misleading use of the verb 'to connect', and considering that we changed bottom-up with 'automatic' and top-down with 'controlled' processing according to Botvnick et al., 2001 and Erb et al., 2016 (see the response to point 4 for details), for the sake of clarity, in the revised version of the manuscript, the sentences were rephrased as follows:*

*'Several neuropsychological studies considered hand movement as a proxy to detect and quantify the intertwined automatic and controlled strategic motor corrections needed to update actions based on cognitive evaluations that rapidly occur during the decision process [17-20, 27-29]'.*

4. Please precisely define your definitions of bottom-up and top-down processes (including “processes”) - these terms are sufficiently equivocal to raise debates (with some scholars questioning their relevance altogether).

*We thank the Reviewer for pointing out this important theoretical issue. In the revised version of the manuscript, we accurately reviewed our wording to be more precise and adherent to the cited literature. In particular, we changed bottom-up with 'automatic' and top-down with 'controlled' processing according to Botvnick et al., 2001 and Erb et al., 2016.*

*In our manuscript, the term automatic response refers to the activation of the direct pathway from a stimulus to its' default response (i.e., the response generated in the absence of any instruction)' (Shenhav et al., 2013, page 219). Conversely, the term controlled response refers to a selection entirely under the control of the observer's intentions, an active, volitional process based on expectancy and goal setting.*

*This theoretical framework is usually employed to study the action selection and conflict processing upon a choice using the MT approach. Indeed, in the recent work by Jonathan Freeman et al. (2020, page 829), entitled: 'Computational approaches to the neuroscience of social perception', it is reported that:*

*'In social psychology, social categorization has traditionally been considered an automatic and obligatory perceptual process that precedes any more 'cognitive' social processes such as stereotyping (Allport, 1954). However, alternative approaches emphasize the idea that social-conceptual knowledge (i.e., stereotypical associations) and other top-down social cognitive processes can weigh in on face processing before a percept has solidified, thereby allowing implicit stereotypes to shape face perception (Freeman and Ambady, 2011; Freeman and Johnson, 2016).'*

5. Please provide a much more rigorous and convincing rationale for you 3-phase choice: this is an important feature of your work that is currently ill-justified -> why are 3 phases needed - compared to 2, 4 or an infinity (at the theoretical level, as this is not a trivial choice for MT)? Indeed, beneath the surface (appealing simplicity), this 3-phase categorization has the potential to jeopardize the very advantages of mouse-tracking (providing continuous signals).

*We want to thank the Reviewer for having raised these questions since we have the opportunity to get into the details of the theoretical and empirical underpinning of the novel phase-based approach developed. For the sake of clarity, we have addressed the Reviewer's questions as follows:*

*First point: "Why are 3 phases needed - compared to 2, 4 or an infinity (at the theoretical level, as this is not a trivial choice for MT)?"*

*The three phases have been selected upon an accurate analysis of the empirical findings of seminal works regarding mouse-tracking and conflict monitoring.*

*Indeed, based on the ‘conflict monitoring hypothesis’ introduced by Botvinick et al., 2001 and on the recent work by Bundt and coworkers (2018), the Initiation Time was selected to capture the ‘Early-Phase’ of the emerged conflict at the basis of the IAT Congruency Effect. Accordingly, we report an extracted part of the manuscript discussion mentioned above, stressing the relevance of the Initiation Times in MT experiments.*

*‘The notion that cognition biases actions after movement initiation is further supported by our finding that compared to relatively slow initiated mouse movements, relatively fast initiated movements deviated more from the optimal trajectory. This suggests that participants experienced a stronger attraction to the alternative response when there was little time to process a stimulus before movement initiation. In addition to the here observed implementation of item-specific control (rather than simple conflict) both before and after movement onset, the current study extends previous work in another way. Most of the previous studies showing that cognition leaks into the response execution interval involved intentional, top-down processes based on the learning and implementation of instructed rules. Conversely, in the present study we demonstrate that incidental, bottom-up control processes that are triggered within the trial (i.e., in an ISPC task, only upon stimulus presentation does the system know the required control demands for the ongoing trial) can also bias responses both before and after movement onset. This differs greatly from, for example, the explicit cues used by Buc Calderon et al. [22] to indicate the probability of an upcoming stimulus. By demonstrating that bottom-up, item-specific control affects mouse trajectories after movement onset, we provide novel insights into the circumstances under which cognitive processing and response execution can temporally overlap’; (Bundt et al., 2018, page 10).*

*Based on the above and others works (Botvinick et al., 2001; Shenhav et al., 2013; Erb et al., 2016), the Early-Phase computed using the Initiation Times (namely, the latency from stimuli presentation and the onset of mouse movements) would mainly correspond the activation of the automatic responses elicited by stimulus processing. According to Erb’s proposal (Erb et al., 2016), the decision conflict between automatic and controlled responses would start here.*

*Regarding the Mid-Phase, the Maximum Deviation Time was selected upon analysing a large pool of Mouse-Tracking literature. In this regard, as reported in the elegant review by Stillman and coworkers (Stillman et al., 2918), the Maximum Deviation Time is the most commonly used metrics in mouse-tracking research quantify the magnitude of response conflict. This measure is computed by comparing the actual trajectory to a straight trajectory from start to response termination, and trajectories that are more similar to the straight trajectory are interpreted as reflecting less conflict between the two options [5]. For instance, the area under the curve (AUC) quantifies response conflict by calculating the area between the actual and the idealized straight trajectory, and the maximum deviation (MD) calculates how far the furthest point on the actual trajectory is from the idealized straight trajectory” (Stillman et al., 2018, page 534). Therefore, the temporal metric related to Maximum Deviation has been selected to compute the ‘Mid-Phase’, thus quantifying the amount of emerged conflict from a temporal point of view.*

*The Late-Phase is completely novel but, as reported in the manuscript, is calculated using well-established MT metrics such as Response Time and Maximum Deviation Time (Stillman et al., 2018; Freeman et al., 2011).*

*In addition, besides the theoretical and empirical support mentioned above, to reassure the Reviewer about the reliability and, most importantly, methodological validity of our phase-based approach, we performed a series of additional analyses using the Area Under the Curve (AUC) metric. From a mathematical point of view, the AUC represents the most suitable MT metric to capture all the ‘infinite’ unfolding phases of MT trajectories. Indeed, AUC is calculated by an integral, thus combining infinitesimal data. In particular, as proof of the validity of our phase-based approach, we explored the relationship between the AUC data with the RT-race-IAT scores. Specifically, we computed the D value as the differences between AUC data collected in stereotypical-congruent vs stereotypical-incongruent trials. The D values were included in a general linear model to test their association with the RT-race-IAT D score.*

***Figure 1.*** *Linear regression between RT-race-IAT D Score vs Δ-AUC; the solid line indicates the best linear model and the dashed lines indicate the 95% confidence intervals.*

*As expected by the PCA findings, the additional analyses showed that the RT-race-IAT D score is a significant predictor of Δ-AUC (Figure 1). Thus, an MT metric capable of capturing the infinite unfolding phases of an MT trajectory as the AUC is associated with the IAT Congruency Effect. However, due to its nature is not capable of following the evolution over time of this Congruency Effect. As demonstrated in our manuscript (please see manuscript Fig.6), the novel phase-based approach allows overcoming this limit obtaining a fine-grained resolution of the unfolding decision conflict in a categorization task.*

*Closing the answer to the first point, with the above explanations and additional analyses, we hope to have made more explicit how the phase-based approach was computed and, most importantly, the theoretical and empirical reliability of the approach.*

*Second point: "Indeed, beneath the surface (appealing simplicity), this 3-phase categorization has the potential to jeopardize the very advantages of mouse-tracking (providing continuous signals)."*

*Regarding this Reviewer’s criticism, in our opinion, ‘simplicity’ is not synonymous with a lack of methodological rigour. Indeed, in light of the theoretical and empirical foundations papers included in the reference list (Botvinick et al., 2001; Shenhav et al., 2013Erb et al., 2016), as well as methodological details provided in the first point of this response; we believe that the appealing simplicity of our phase-based approach is a strength that will help the implementation of this new tool for boosting the resolution analysis of the Congruency Effect in a categorisation task. Henceforth, taking into account the methodological reliability of our phase-based approach, we think that its adoption will potentiate rather than jeopardy the MT approach by capturing not only the amount of conflict but even how this conflict has unfolded over time. This latter aspect will provide a temporal dissection of the conflict that emerged upon a choice according to established and recognized cognitive models concerning conflict monitoring (Botvinick et al., 2001; Shenhav et al., 2013; Erb et al., 2016).*

*To conclude, if the Reviewer thinks that the above explanations and the additional analyses will help make our materials and methods more precise regarding the phase-based approach computation, we could add them in the supplementary materials.*

6. Stating that the "early phase (...) [may comprise] purely implicit, bottom-up mechanisms" is currently inappropriate. Please refer to recent papers discussing the problems associated with clearly defining purely "implicit" processes. Second, by no means should implicit be equated with bottom-up processes (as I understand them but, again, these need to be defined). Third, past research relies on mouse-tracking to question the implicit/explicit distinction and some of the study’s results go in the same direction. So, to avoid incorrectness/overstatement, please stay close in your wording to what this early phase may plausibly reflect.

*We thank the Reviewer for raising a potential terms’ inconsistency concerning implicit/explicit processing. Thus, for the sake of consistency, in the revised version of the manuscript, the discussion of Early Phase was rephrased as follows:*

*‘On each trial, participants clicked a ‘start’ button appearing at the bottom centre of the screen, and the target stimulus appeared in its place; from that moment, an automatic default response is predominantly activated in line with the ‘direct pathway’ suggested by Botvinick, Braver (27) and Erb, Moher (29). The latency between stimulus presentation and actual mouse movement was considered the first temporal interval, namely the Early Phase.’*

7. As the terms conscious and unconscious processes are often debated, either they need to be defined or should be replaced (I am for instance doubting that JB Freeman uses the conscious/unconscious terminology, except maybe in some of his early work). Later on, the authors use "unconscious, purely implicit, bottom up processes". I urge the authors to be both more cautious and precise in their wording. They are not equivalent according to current literature and are equivocal without further clarification.

*Similarly to point 6, again, we thank the Reviewer for raising a potential terms’ inconsistency. Accordingly, we have promptly revised all the parts in the manuscript to avoid this inconsistency especially concerning the potential misleading overlapping of unconscious/implicit/bottom-up processing.*

8. I would have expected a better integration of this bimodality/unimodality aspects with the authors' phases model. Could the authors please provide some insights?

*The distributional analysis of MT trajectories was crucial for our experimental analysis. Indeed, by the shape analysis, we have shown that even in a race-IAT adopting the MT approach, a dynamic process model can best account for the data, assuming simultaneous co-activation of competing stereotypes that gradually get resolved over time. Notably, these findings are in line with previous works with similar experimental settings (Yu et al., 2012), and most importantly, they have been prodromal to developing our phase-based approach.*

*In this regard, as shown in the above figure extracted by the seminal work by Jonathan Freeman and Rick Dale entitled: ‘Assessing bimodality to detect the presence of a dual cognitive process’, the distributional analyses of the MT trajectories are usually employed to assess the existence of two different types of distribution. Namely a monomodal (upper part of the figure) or a bimodal (middle and bottom part of the figure) distribution.*

*The bimodal distributions of the MT trajectories provide a precise check-point analysis, specifically the Maximum Deviation, to explore automatic vs controlled influence. Conversely, the unimodal distributions by not providing a clear check-point strengthen the need for developing novel tools that can dissect and weigh this dynamical automatic and controlled processes interaction over time. Therefore, we developed our phase-based approach.*

*This reasoning was reported in the following part of the manuscript discussion (page 21, rows 533-556):*

*‘Traditionally, the MT approach has always been used to rule out which class of cognitive frameworks has been engaged in guidance the temporal evolution of judgements and decisions [26]. The dual-system or stage-based perspective is the most widely endorsed theoretical framework used to make predictions about the temporal sequence of a decision process towards a target choice [33, 34]. This framework implies that a decision unfolds via a two-system sequence whereby a faster, automatic driven representation (system 1) guide the early path of hand movement towards the target choice, and, after a time lag, a slower cognitive system (system 2) can exert a controlled response to correct this initial response [15, 22, 26-29, 33, 34, 65]. Besides the dual-system perspective, another class of theoretical model refers to a dynamical competition (Dynamical Framework) of different controlled and automatic representations that synchronously interact in guiding the path of hand movement towards the target choice [27-29, 35, 36, 66]. Notably, the distributional analyses of the MT metrics allow detecting whether the trajectories follow an abrupt profile, characterised by a sharp mid-flight correction mirroring the competition between two systems during the evolution of the movement (Dual-system framework; [33, 34]), or relatively smooth profile mirroring a dynamical competition of different controlled and automatic processes that simultaneously compete in guiding the movement (Dynamical framework; [35, 36, 66]). In this respect, in accordance with previous findings by Yu, Wang (31) gathered in a different not race IAT structure, our distributional analyses showed that in both congruent and incongruent trials, mouse trajectories displayed smooth fashion profile indicating that, overall, in a race IAT structure, the decision process and the consequently raised cognitive conflict appear to unfold guided by dynamical cognitive processing (see Fig. 4).*

*This latter finding is also in line with the ‘conflict monitoring hypothesis’ [27-29] strengthening the need to better characterize this continuous monomodal dynamical competition by studying its unfolding and identifying behavioural markers of the imbalance between automatic and controlled processes over time. Accordingly, our novel phase-based approach allowed us to temporally dissect this continuous decision conflict by combining different temporal MT-metrics (IT, MD-Time and RT)’.*

*With the above explanations we hope of having made clearer how the distributional analysis has been integrated in the manuscript.*

*In addition, the Reviewer may want to consider that this response could even strengthen the one reported in point 5 regarding the potential risk to jeopardize the very advantages of mouse-tracking (providing continuous signals) employing our phase-based approach. Indeed, as reported above, classically with a bimodal distribution, the MT trajectories are dissected at least in two parts (depending on the inflection points) despite a starting continuous signal. In this line of reasoning, once again, the adoption of our phase-based approach will potentiate rather than jeopardy the existing methods, such as distributional analyses, to dissect the continuous signal provided by the MT approach to explore how a decision conflict unfolded over time mainly when this conflict emerged by a dynamical competition (unimodal distribution) as in our work.*

9. It seems like the authors did not counterbalance critical congruent and incongruent blocks. If not, this is a serious issue, both experimentally (problem in design) and statistically (incorrect estimation of effect sizes - here, artificially increasing effect sizes). Could the authors please clarify and provide appropriate justification?

*Concerning the counterbalance of blocks, we are fully aware that an order effect caused by cognitive inertia may affect the IAT effect in a between-subject experiment, thus representing a severe issue both experimentally and statistically. However, taking into account the primary aim of our study and, most importantly, the within-subject experimental design, compelling evidence (Nosek, Greenwald and Banaji, 2005; Greenwald et al., 2021) has clearly shown that a lack of counterbalancing IAT blocks does not seriously impact on experimental findings.*

*In this regard, the Reviewer may want to note that our main porpoise when designing the experiment was to compare a well-established and recognized stimuli-association reaction time test directly with a one with the same structure incorporating mouse movements tracking. In this line of reasoning, the standard race-implicit association test (Greenwald, Nosek, Banaji, 2003) has been chosen as a ‘model’ to carry out this comparison and to develop a new phase-based approach obtaining a fine-grained resolution of the unfolding decision conflict in a categorization task. In other words, the main aim of our study was not to measure implicit racial bias in Italian students but rather to compare two parallel forms of the same tests. Our study's explicit goals were to first evaluate whether we could observe a similar congruency effect with an MT approach; second, to identify the best MT-metrics to capture the congruency effect with this approach. Finally, to explore whether a phase-based approach could dissect the congruency effect's continuous unfolding for the first time.*

*Moreover, regarding the Reviewer’s concern, if on one side, an order effect could affect IAT scores, on the other side, the influence of this effect has been proven to be small (Nosek, Greenwald and Banaji in 2005). Indeed, as shown in figure 2 by Nosek, Greenwald and Banaji (2005), the magnitude of the pairing order effect for the unbalanced black and white IAT with 20 trials (as employed in our study) is smaller than .10.*

***Nosek, Greenwald and Banaji (2005) Figure 2.*** *Magnitude of the pairing order effect by the number of response trials in the reverse single discrimination block for Black-White, Old-Young, Gender-Science, Asian-White, and Dark Skin-Light Skin Implicit Association Tests (IATs). A value of zero indicates the absence of a pairing order effect (Study 4).*

*In addition, Greenwald et al. (2021, explicitly stated that although counterbalancing is recommended as a general good practice, ‘the effect of order of combined tasks is typically small, meaning (statistically) that its effect on correlations with other variables is likely to be very small, possibly unnoticeable’ (Greenwald et al., 2021, page 8 of the online PDF-version). Besides, the authors suggested that ‘from using the counterbalancing […] sample means will not be displaced due to the chosen order of combined tasks influencing the mean IAT value for the entire sample’.*

*Nevertheless, the Reviewer may want to consider that we are not interested in a between-groups or stimuli categories comparison. We aimed at correlating the congruency effects extracted from the classic and MT approaches.*

*Regardless, since we are fully aware that the Reviewer’s concern regarding the order effect must be addressed, we have discussed this potential bias in the limitation section of the revised manuscript. Accordingly, in the revised manuscript it is now reported:*

*‘In addition, since we decided to use a classical within-subject design to directly compare participants’ performance gathered in the classical RT-race-IAT with the MT one, we did not counterbalance the temporal order of the congruent and incongruent combined tasks. It is worth noting that an order effect caused by cognitive inertia may affect the IAT effect, especially in a between-subject design [65, 66]. Therefore, future studies that aim to employ our phase-based approach in a between-subject paradigm should consider counterbalancing the order of the experimental blocks, thus mitigating the potential order effect (for details concerning the best research practices for using the IAT see [65])’.*

10. Why only subject ID, and as random intercept only (what about slopes?) and what about stimuli? Please provide a strong justification of your selected model and, if changes in random effects impact your main findings, this should be acknowledged.

*We adopted random intercept to overcome the non-independence of a subject's residuals' issue typical of within-subjects analysis. However, we believe that our design is not suitable for introducing random slope for two reasons. First, we could have added a random slope for the item, but we do not have any reason to believe that, for example, picture A would be associated with a different slope from picture B. Secondly, we wanted to keep the computation of the congruency effect as more straightforward as possible to be compared with the classic IAT D-Score, in which, by definition, the effect of items had never been considered.*

*Finally, we thank the Reviewer for all the efforts made to improve our manuscript and for the attention given to our work. We believe in having addressed all the Reviewer’s requests and criticisms with the above point-to-point response.*

***Essential references Reviewer 1***

- *Botvinick, M. M., Braver, T. S., Barch, D. M., Carter, C. S., & Cohen, J. D. (2001). Conflict monitoring and cognitive control. Psychological review, 108(3), 624.*
- *Brooks, J. A., Stolier, R. M., & Freeman, J. B. (2021). Computational approaches to the neuroscience of social perception. Social cognitive and affective neuroscience, 16(8), 827–837. https://doi.org/10.1093/scan/nsaa127.*
- *Bundt, C., Ruitenberg, M., Abrahamse, E. L., & Notebaert, W. Early and late indications of item-specific control in a Stroop mouse tracking study. PloS one, 13(5), e0197278.* [*https://doi.org/10.1371/journal.pone.0197278*](https://doi.org/10.1371/journal.pone.0197278) *(2018).*
- *Cohen, J. Statistical power analysis for the behavioral sciences. (L. Erlbaum Associates, 1988).*
- *Erb, C. D., Moher, J., Sobel, D. M., & Song, J. H. (2016). Reach tracking reveals dissociable processes underlying cognitive control. Cognition, 152, 114-126.*
- *Freeman, J. B., & Dale, R. (2013). Assessing bimodality to detect the presence of a dual cognitive process. Behavior research methods, 45(1), 83–97.* [*https://doi.org/10.3758/s13428-012-0225-x*](https://doi.org/10.3758/s13428-012-0225-x)*.*
- *Freeman, J. B., Dale, R., & Farmer, T. A. Hand in motion reveals mind in motion. Frontiers in psychology, 2, 59.* [*https://doi.org/10.3389/fpsyg.2011.00059*](https://doi.org/10.3389/fpsyg.2011.00059) *(2011).*
- *Gorsuch, R. L. Factor Analysis (1983).*
- *Greenwald, A. G., Brendl, M., Cai, H., Cvencek, D., Dovidio, J. F., Friese, M., Hahn, A., Hehman, E., Hofmann, W., Hughes, S., Hussey, I., Jordan, C., Kirby, T. A., Lai, C. K., Lang, J., Lindgren, K. P., Maison, D., Ostafin, B. D., Rae, J. R., Ratliff, K. A., … Wiers, R. W. Best research practices for using the Implicit Association Test. Behavior research methods, 10.3758/s13428-021-01624-3. Advance online publication.* [*https://doi.org/10.3758/s13428-021-01624-3*](https://doi.org/10.3758/s13428-021-01624-3) *(2021).*
- *Greenwald, A. G., Nosek, B. A., & Banaji, M. R. Understanding and using the implicit association test: I. An improved scoring algorithm. Journal of personality and social psychology, 85(2), 197–216.* [*https://doi.org/10.1037/0022-3514.85.2.197*](https://doi.org/10.1037/0022-3514.85.2.197) *(2003).*
- *Nosek, B. A., Greenwald, A. G., & Banaji, M. R. Understanding and using the Implicit Association Test: II. Method variables and construct validity. Personality & social psychology bulletin, 31(2), 166–180.* [*https://doi.org/10.1177/0146167204271418*](https://doi.org/10.1177/0146167204271418) *(2005).*
- *Osborne, J. W., Costello, A. B. J. P. a., research & evaluation. Sample size and subject to item ratio in principal components analysis. 9, 8 (2004).*
- *Shenhav, A., Botvinick, M. M., & Cohen, J. D. (2013). The expected value of control: an integrative theory of anterior cingulate cortex function. Neuron, 79(2), 217-240.*
- *Stillman, P. E., Shen, X., & Ferguson, M. J. How Mouse-tracking Can Advance Social Cognitive Theory. Trends in cognitive sciences, 22(6), 531–543.* [*https://doi.org/10.1016/j.tics.2018.03.012*](https://doi.org/10.1016/j.tics.2018.03.012) *(2018).*
- *Yu, Z., Wang, F., Wang, D. & Bastin, M. Beyond reaction times: Incorporating mouse-tracking measures into the implicit asociation test to examine its underlying proces. Social Cognition 30, 289-306, doi:10.1521/soco.2012.30.3.289 (2012).*

**Reviewer #2**

1. Regarding the utility of Mouse Tracking for studying race biases, in addition to the citations included in the paper, Melnikoff et al., 2020 showed Mouse-Tracking are useful in studying racial biases. It predicted behavioral racial biases. Although it did not compare IAT test and Mouse-Tracking directly, it makes me wonder what is new about this manuscript. There are a lot of descriptive results in the manuscript, but it’s unclear to me what is the main question this research is trying to solve. This is partly due to my questions (see below) about the two main points the authors are trying to demonstrate in the paper.

*In what follows, we would like to stress the novelty of our manuscript as we believe that the Reviewer may have underestimate it in the original version of the manuscript. In our opinion, indeed, the manuscript by Melnikoff et al. would strengthen rather than weaken the novelty of our work by stressing the need for a novel analysis tool of the MT data at the service of characterization of racial biases.*

*In this regard, the Reviewer may want to note that in the manuscript by Melnikoff et al. (Melinkoff et al., 2021), the authors demonstrated that the MT approach is an effective tool to capture conflict in explicit evaluations of racial groups. Indeed, the authors measured the racial bias by employing an MT task profoundly different from the race-IAT paradigm and, most importantly, an MT task specifically designed to explore explicit racial evaluations. Accordingly, they adopted the procedure developed by Wojnowicz and colleagues (Wojnowicz et al., 2009) involving Black/White names and positive/negative concepts as stimuli, with the response options ‘Like’ or ‘Dislike’. Participants were called to respond ‘Like’ to Black and White people’s names and exhibited larger AUCs in responding ‘Like’ to Black names than White ones.*

*Thus, for the following reasons:*

1. *racial bias measured by an MT task specifically designed to explore explicit racial evaluations and, henceforth, profoundly different from the race-IAT paradigm employed in our experimental work;*
2. *the lack of a direct comparison of the employed MT task with the gold standard tool for measuring racial bias (namely the Race-Implicit Association Test) as we did in our experimental work;*
3. *the use of only the Area Under the Curve data to measure the racial bias, instead of the use of a comprehensive Principal Component Analysis to identify which MT metrics were the more suitable and accurate from a statistical point of view to measure racial bias as we did;*
4. *the lack of any distributional analyses of the MT data able to shed light on the cognitive processes engaged during the explicit racial evaluations made by participants;*
5. *the lack of testing a new tool to follow the temporal unfolding of the decision conflict as we did by employing the phase-based approach;*

*We believe that the work by Melinkoff and colleagues (Melinkoff et al., 2021) did not weaken the novelty of our manuscript.*

*The above reported reasoning was inserted in the following part of the revised manuscript introduction (page 6, rows 140-148):*

*‘In this regard, recently, Melnikoff and colleagues [37] demonstrated that the MT approach is an effective tool to capture conflict in explicit evaluations of racial groups. However, the authors measured the racial bias by employing an MT task profoundly different from the race-IAT paradigm [38]. Accordingly, they adopted the procedure developed by Wojnowicz and colleagues [38] involving Black/White names and positive/negative concepts as stimuli, with the response options 'Like' or 'Dislike'. Participants were called to respond 'Like' to Black and White people's names and exhibited larger AUCs in responding 'Like' to Black names than White ones [37, 38]. The dissimilarity of the MT approach used by Melnikoff and colleagues [37] compared with an IAT structure makes the adoption of the MT approach in the classical race-IAT of primary interests’*.

2. In the intro and discussion, the authors mentioned multiple times about bottom-up and top-down processes and seem to suggest that Mouse-Tracking results can help to adjudicate the different processes. This is also one of the main goals of the paper. Maybe I misunderstand the authors intention, but I’m a bit confused about this theoretical point. What are top-down and bottom-up conflict, respectively in the Mouse-Tracking context? And how and what question is the three phases model can help to solve?

*We thank the Reviewer for pointing out this important theoretical issue. This criticism was also one of Reviewer 1’s concerns. For this reason, in the revised version of the manuscript, we accurately reviewed our wording to be more precise and adherent to the cited literature. In particular, for the sake of clarity, we changed bottom-up with 'automatic' and top-down with 'controlled' processing according to Botvnick et al., 2001 and Erb et al., 2016.*

*In our manuscript, the term ‘automatic response’ refers to the activation of the direct pathway from a stimulus to its' default response (i.e., the response generated in the absence of any instruction)' (Shenhav et al., 2013, page 219). Conversely, the term ‘controlled responses’ refers to a selection entirely under the control of the observer's intentions, an active, volitional process based on expectancy and goal setting.*

*This theoretical framework is usually employed to study the action selection and conflict processing upon a choice using the MT approach.*

3. Could the authors help me to understand the reasons behind dissecting by temporal mark? Also, after the factor analysis, the authors picked one metric from each factor to follow up. However, the other unpicked metrics show similar quality to the picked ones. What would the results be if the other metrics from the two factors are used? This makes me question whether the second goal of this paper, identify metrics from Mouse-Tracking that is comparable to the IAT metrics, is achieved properly.

*We want to thank the Reviewer for having raised these questions since we have the opportunity to get into the details of the theoretical and empirical underpinning of the novel phase-based approach developed and the PCAs analyses computed. For the sake of clarity, we have addressed the Reviewer's questions as follows:*

*First point: "Could the authors help me to understand the reasons behind dissecting by temporal mark?"*

*The need for a temporal dissection to explore the imbalance between automatic and controlled processes engaged upon a raised decision conflict during a categorisation task by employing the phase-based approach is intimately related to our distributional analyses findings.*

*Accordingly, the distributional analysis of MT trajectories was a turning point of our experimental analyses. Indeed, by the shape analysis, we have shown that even in a race-IAT adopting the MT approach, a dynamic process model can best account for the data, assuming simultaneous co-activation of competing stereotypes that gradually get resolved over time. Notably, these findings are in line with previous works with similar experimental settings (Yu et al., 2012), and most importantly, they have been prodromal to developing our phase-based approach.*

*In this regard, as shown in the above figure extracted by the seminal work by Jonathan Freeman and Rick Dale entitled: ‘Assessing bimodality to detect the presence of a dual cognitive process’, the distributional analyses of the MT trajectories are usually employed to assess the existence of two different types of distribution. Namely a monomodal (upper part of the figure) or a bimodal (middle and bottom part of the figure) distribution.*

*The bimodal distributions of the MT trajectories provide a precise check-point analysis, specifically the Maximum Deviation, to explore automatic vs controlled influence. Conversely, the unimodal distributions by not providing a clear check-point strengthen the need for developing novel tools that can dissect and weigh this dynamical automatic and controlled processes interaction over time. Therefore, we developed our phase-based approach.*

*This reasoning was reported in the following part of the manuscript discussion (page 21, rows 533-556):*

*‘Traditionally, the MT approach has always been used to rule out which class of cognitive frameworks has been engaged in guidance the temporal evolution of judgements and decisions [26]. The dual-system or stage-based perspective is the most widely endorsed theoretical framework used to make predictions about the temporal sequence of a decision process towards a target choice [33, 34]. This framework implies that a decision unfolds via a two-system sequence whereby a faster, automatic driven representation (system 1) guide the early path of hand movement towards the target choice, and, after a time lag, a slower cognitive system (system 2) can exert a controlled response to correct this initial response [15, 22, 26-29, 33, 34, 65]. Besides the dual-system perspective, another class of theoretical model refers to a dynamical competition (Dynamical Framework) of different controlled and automatic representations that synchronously interact in guiding the path of hand movement towards the target choice [27-29, 35, 36, 66]. Notably, the distributional analyses of the MT metrics allow detecting whether the trajectories follow an abrupt profile, characterised by a sharp mid-flight correction mirroring the competition between two systems during the evolution of the movement (Dual-system framework; [33, 34]), or relatively smooth profile mirroring a dynamical competition of different controlled and automatic processes that simultaneously compete in guiding the movement (Dynamical framework; [35, 36, 66]). In this respect, in accordance with previous findings by Yu, Wang (31) gathered in a different not race IAT structure, our distributional analyses showed that in both congruent and incongruent trials, mouse trajectories displayed smooth fashion profile indicating that, overall, in a race IAT structure, the decision process and the consequently raised cognitive conflict appear to unfold guided by dynamical cognitive processing (see Fig. 4).*

*This latter finding is also in line with the ‘conflict monitoring hypothesis’ [27-29] strengthening the need to better characterize this continuous monomodal dynamical competition by studying its unfolding and identifying behavioural markers of the imbalance between automatic and controlled processes over time. Accordingly, our novel phase-based approach allowed us to temporally dissect this continuous decision conflict by combining different temporal MT-metrics (IT, MD-Time and RT)’.*

*Thus, once clarified the reason behind the need of the phase-based approach to temporally dissect this monomodal continuous decision conflict, the three phases have been selected upon an accurate analysis of the empirical findings of seminal works regarding mouse-tracking and conflict monitoring.*

*Indeed, based on the ‘conflict monitoring hypothesis’ introduced by Botvinick et al., 2001 and on the recent work by Bundt and coworkers in 2018 on PlosOne Journal, the Initiation Time was selected to capture the ‘Early-Phase’ of the emerged conflict at the basis of the IAT Congruency Effect. Accordingly, we report an extracted part of the manuscript discussion mentioned above, stressing the relevance of the Initiation Times in MT experiments.*

*‘The notion that cognition biases actions after movement initiation is further supported by our finding that compared to relatively slow initiated mouse movements, relatively fast initiated movements deviated more from the optimal trajectory. This suggests that participants experienced a stronger attraction to the alternative response when there was little time to process a stimulus before movement initiation. In addition to the here observed implementation of item-specific control (rather than simple conflict) both before and after movement onset, the current study extends previous work in another way. Most of the previous studies showing that cognition leaks into the response execution interval involved intentional, top-down processes based on the learning and implementation of instructed rules. Conversely, in the present study we demonstrate that incidental, bottom-up control processes that are triggered within the trial (i.e., in an ISPC task, only upon stimulus presentation does the system know the required control demands for the ongoing trial) can also bias responses both before and after movement onset. This differs greatly from, for example, the explicit cues used by Buc Calderon et al. [22] to indicate the probability of an upcoming stimulus. By demonstrating that bottom-up, item-specific control affects mouse trajectories after movement onset, we provide novel insights into the circumstances under which cognitive processing and response execution can temporally overlap’; (Bundt et al., 2018, page 10).*

*Based on the above and others works (Botvinick et al., 2001; Shenhav et al., 2013; Erb et al., 2016), the Early-Phase computed using the Initiation Times (namely, the latency from stimuli presentation and the onset of mouse movements) would mainly correspond the activation of the automatic responses elicited by stimulus processing. According to Erb’s proposal (Erb et al., 2016), the decision conflict between automatic and controlled responses would start here.*

*Regarding the Mid-Phase, the Maximum Deviation Time was selected upon analysing a large pool of Mouse-Tracking literature. In this regard, as reported in the elegant review by Stillman and coworkers entitled ‘How Mouse-tracking Can Advance Social Cognitive Theory’: ‘Quantifying Conflict. The most commonly used metrics in mouse-tracking research quantify the magnitude of response conflict between the choice outcomes by gauging the relative directness with which participants make their response. This is done by comparing the actual trajectory to a straight trajectory from start to response termination, and trajectories that are more similar to the straight trajectory are interpreted as reflecting less conflict between the two options [5]. For instance, the area under the curve (AUC) quantifies response conflict by calculating the area between the actual and the idealized straight trajectory, and the maximum deviation (MD) calculates how far the furthest point on the actual trajectory is from the idealized straight trajectory” (Stillman et al., 2018, page 534), the most suitable MT metric to quantify the amount of generated conflict is the Maximum Deviation. Therefore, the temporal metric related to Maximum Deviation has been selected to compute the ‘Mid-Phase’, thus quantifying the amount of emerged conflict from a temporal point of view.*

*The Late-Phase is completely novel but, as reported in the manuscript, is calculated using well-established MT metrics such as Response Time and Maximum Deviation Time (Stillman et al., 2018; Freeman et al., 2011).*

*Closing the answer to the first point, with the above explanations, we hope to have made more explicit how the distributional analysis was intimately related to the phase-based approach, how the phase-based approach was computed and, most importantly, the theoretical and empirical reliability of the approach.*

*Second point: "Also, after the factor analysis, the authors picked one metric from each factor to follow up. However, the other unpicked metrics show similar quality to the picked ones. What would the results be if the other metrics from the two factors are used? This makes me question whether the second goal of this paper, identify metrics from Mouse-Tracking that is comparable to the IAT metrics, is achieved properly."*

*As stated by Greenwald and colleagues (2003) and further supported (ex. Nosek et al., 2014; Greenwald et al., 2021), the computation of the D score for the classical version of the Implicit Association Test provides a reliable and straightforward measure of implicit bias. The D score represents, thus, the best-performing measures when compared to the other four emerging from the latencies’ transformations (see Greenwald et al., 2003, study 2) and, consequently, is widely used as a univocal measure of implicit bias.*

*When considering the MT-IAT, things get more complicated since the hand movement generates several metrics that can be considered and tested, conducing to different and possibly inconsistent results.*

*By performing a PCA, we made an effort to clarify what metrics should be considered for calculating the implicit bias and, above all, what latent constructs are involved in the MT-IAT. This approach was employed to adequately establish relations between variables avoiding fishing, i.e., to test all variables to find some significant results, the well-known problem of Bonferroni's multiple comparisons (see Boston et al., 1991).*

*In this regard, by identifying the two main factors underlying the hand movement during a decisional process, namely a 'geometrical component' (kinematic-related) and a 'temporal' one, we gave, in our opinion, a relevant contribution to the understanding of cognitive processes at the basis of this tasks, something relevant from a psychological and theoretical point of view, and reassuring the construct validity, something relevant from a statistical point of view.*

*In addition, to test the variable with the highest saturation score of each item, instead of all the variables, was done precisely to avoid fishing and obtain a more straightforward way to look at the MT-IAT data.*

*As far as the variables with the highest saturations scores represent the variables with the higher regression coefficient (Gallucci, Leone & Berlingeri, 2017; Field et al., 2012) and, thus, with the highest contribution to each factor, it is logical to assume that they are very representative of the latent construct of interest. For this reason, Spearman (1904a, 1904b) suggests using the criteria of saturation scores for interpreting the psychological mechanisms underlying the factors. By following this way of reasoning, considering that the PCA, as all the methods included in factor analysis, starts from the correlation matrix, we assume that the other metrics not tested would lead to similar results of those correlated. This latter aspect would, by definition, imply that variables included in a single factor are correlated and, thus, cannot be included in a statistical model avoiding the risk of collinearity (Field et al., 2012; Mason & Perreault, 1991).*

*We are aware that the issue mentioned above is often solved by calculating a factorial score (usually based on regression) for each construct to insert in the statistical models. However, computation of a factorial score was not feasible in our case due to the need to calculate PCAs for each condition and the consequent number of variables to consider. Even in the case of factorial scores, the variables with the highest saturation would contribute to the computation of scores without altering the results we have reported in the current study.*

*This latter aspect is even more valid considering the manuscript Table 4, in which it emerged that the selection of the variables was made concerning the highest degree of saturation, i.e., of contribution to the single factor, and communality, i.e., the of contribution to the whole factorial structure.*

*According to all these considerations, we assume the more economical but rigorous way to achieve the goal mentioned by the Reviewer, namely, to identify metrics from Mouse-Tracking that are comparable to the IAT metrics. This achievement is further supported by our data and, in particular, by the correlation between our metrics and the IAT D score.*

*We hope that the Reviewer, once considering all these statistical aspects, would reconsider the value of our procedure.*

4. One thing Mouse-Tracking is inherently different from IAT is that people are explicitly evaluate the targets in Mouse-Tracking (more like self-report). In a standard Mouse-Tracking task, people can take their time to reach a final decision and this is part of the RT data. However, IAT sometimes pose a time limit on responses or take care of the longer time when calculating D score. Does the authors take some steps in the work to alter the design of the Mouse-Tracking task to the IAT test?

*Actually, we were fully aware of the dissimilarity between the RT- and MT-race-IAT especially concerning the accuracy and response time. Indeed, the Reviewer may want to note that we reported these differences in the results section of the manuscript at page 15 (rows 388-396) as follows:*

*‘****RTs and error rates comparison between RT- and MT-race-IAT.*** *We expected that participants would complete the MT-race-IAT with a higher accuracy, but with longer reaction times as compared to the RT one, due to all the strategic motor corrections engaged during the temporal unfolding of the movements [17-20]. To test this hypothesis, a series of Wilcoxon’s signed-sum test was used to compute differences in both accuracy and RTs between RT- and MT-race-IAT. Results showed a significant higher total accuracy (W = 133.5, p < .001, r = −.575) and longer RTs (W = 966, p < .001, r = −.448) in the MT-race-IAT compared to RT one (Table 4). This was the case in both stereotype-congruent [Accuracy (W = 163., p < .001, r = −.547); RTs (W = 1114, p < .001, r = −.412)] and incongruent trials [Accuracy (W = 521.5, p < .001, r = −.511); RTs (W = 960, p < .001, r = −.449); Table 2]’.*

*We reported all the data concerning accuracy and response times comparisons between RT- and MT-race-IAT in Table 2 of the manuscript.*

*Moreover, it is worth noting that the above-reported differences were obtained after all the processing steps of the MT data, which comprised even the removal of all correct trials with trajectories that were ± 3 standard deviations from the mean on reaction times or with an initialisation time longer than 500 ms. All the MT data processing steps were reported in the Methods section of the manuscript on page 12 (rows 281-307).*

*Thus, considering the above dissimilarity and for the sake of methodological consistency, we did not attempt to alter the MT-race-IAT for calculating a D score from the accuracy and response times gathered by the MT data. We instead decided to preserve the peculiarity of the MT approach and perform a Principal Component Analysis of all MT metrics to identify those more suitable to be associated with the classical RT-race-IAT D scores.*

*Conclusively, we thank the Reviewer for all the efforts made to improve our manuscript and for the attention given to our work. We believe in having addressed all the Reviewer’s requests and criticisms with the above point-to-point response.*

***Essential references Reviewer 2***

- *Boston, J. R., Rudy, T. E., & Kubinski, J. A. (1991). Multiple statistical comparisons: Fishing with the right bait. Journal of critical care, 6(4), 211-220.*
- *Botvinick, M. M., Braver, T. S., Barch, D. M., Carter, C. S., & Cohen, J. D. (2001). Conflict monitoring and cognitive control. Psychological review, 108(3), 624.*
- *Brooks, J. A., Stolier, R. M., & Freeman, J. B. (2021). Computational approaches to the neuroscience of social perception. Social cognitive and affective neuroscience, 16(8), 827–837. https://doi.org/10.1093/scan/nsaa127.*
- *Bundt, C., Ruitenberg, M., Abrahamse, E. L., & Notebaert, W. Early and late indications of item-specific control in a Stroop mouse tracking study. PloS one, 13(5), e0197278.* [*https://doi.org/10.1371/journal.pone.0197278*](https://doi.org/10.1371/journal.pone.0197278) *(2018).*
- *Cohen, J. Statistical power analysis for the behavioral sciences. (L. Erlbaum Associates, 1988).*
- *Erb, C. D., Moher, J., Sobel, D. M., & Song, J. H. (2016). Reach tracking reveals dissociable processes underlying cognitive control. Cognition, 152, 114-126.*
- *Field, A., Miles, J., & Field, Z. (2012). Discovering statistics using R. Sage publications.*
- *Freeman, J. B., & Dale, R. (2013). Assessing bimodality to detect the presence of a dual cognitive process. Behavior research methods, 45(1), 83–97.* [*https://doi.org/10.3758/s13428-012-0225-x*](https://doi.org/10.3758/s13428-012-0225-x)*.*
- *Gallucci, M., Leone, L., & Berlingeri, M. (2017). Modelli Statistici Per le Scienze Sociali. Ediz. Mylab. Con eText. CON Aggiornamento Online.*
- *Greenwald, A. G., Brendl, M., Cai, H., Cvencek, D., Dovidio, J. F., Friese, M., ... & Wiers, R. W. (2021). Best research practices for using the Implicit Association Test. Behavior research methods, 1-20.*
- *Mason, C. H., & Perreault Jr, W. D. (1991). Collinearity, power, and interpretation of multiple regression analysis. Journal of marketing research, 28(3), 268-280.*
- *Melnikoff, D. E., Mann, T. C., Stillman, P. E., Shen, X., & Ferguson, M. J. (2021). Tracking prejudice: A mouse-tracking measure of evaluative conflict predicts discriminatory behavior. Social Psychological and Personality Science, 12(2), 266-272.*
- *Nosek, B. A., Bar-Anan, Y., Sriram, N., Axt, J., & Greenwald, A. G. (2014). Understanding and using the brief implicit association test: Recommended scoring procedures. PloS one, 9(12), e110938.*
- *Shenhav, A., Botvinick, M. M., & Cohen, J. D. (2013). The expected value of control: an integrative theory of anterior cingulate cortex function. Neuron, 79(2), 217-240.*
- *Spearman, C. (1904a). ‘General intelligence’, objectively determined and measured. American Journal of Psychology, 15, 201–293.*
- *Spearman, C. (1904b). The proof and measurement of the association between two things. American Journal of Psychology, 15, 72–101.*
- *Yu, Z., Wang, F., Wang, D. & Bastin, M. Beyond reaction times: Incorporating mouse-tracking measures into the implicit asociation test to examine its underlying proces. Social Cognition 30, 289-306, doi:10.1521/soco.2012.30.3.289 (2012).*
