# Supplementary material for: The biased hand. Mouse-tracking metrics to examine the conflict processing in a race-implicit association test
Source: PLoS One. 2022 Jul 27;17(7):e0271748. doi: 10.1371/journal.pone.0271748 (PMC9328548; doi:10.1371/journal.pone.0271748)
Supplement: Attachment — Submitted filename: Di Palma et al - Response to Additional Editor Comments.docx [file pone.0271748.s003.docx]

First of all, we would like to thank the Editor for appreciating our work, and for all the efforts made in trying to improve it. In what follows, we address the Editor’s concern and report the changes made to the manuscript's revised version.

**Additional Editor comments:**

- Thank you for the revised manuscript. Unfortunately, the original reviewers were not available anymore to evaluate whether your revisions met their requirements. I have reviewed the revised manuscript myself: with the implemented changes it meets the publication requirements with regards to the manuscript. However, without access to the original raw data (so not the results that are in the manuscript and supplements) I cannot accept this manuscript. Please make sure that the original datafiles on which your analyses are based are available and accessible (obviously after they are anonymised). If that is taken care off, I can accept the manuscript as it is.

*As requested, the raw experimental data have been registered at the Open Science Framework platform and are available at the following link:* [*https://osf.io/B2JC5/*](https://osf.io/B2JC5/). *In the revised version of the manuscript, the Data availability statement was changed accordingly. Finally, all the revisions were highlighted in yellow to be easily detected.*

*Conclusively, we thank the Editor for all the efforts to improve our manuscript and the attention given to our work. We believe in having addressed the Editor’s requests with the above response.*
